# A collection of enhancer trap insertional mutants for functional genomics in tomato

**DOI:** 10.1111/pbi.12728

**Published:** 2017-04-20

**Authors:** Fernando Pérez‐Martín, Fernando J. Yuste‐Lisbona, Benito Pineda, María Pilar Angarita‐Díaz, Begoña García‐Sogo, Teresa Antón, Sibilla Sánchez, Estela Giménez, Alejandro Atarés, Antonia Fernández‐Lozano, Ana Ortíz‐Atienza, Manuel García‐Alcázar, Laura Castañeda, Rocío Fonseca, Carmen Capel, Geraldine Goergen, Jorge Sánchez, Jorge L. Quispe, Juan Capel, Trinidad Angosto, Vicente Moreno, Rafael Lozano

**Affiliations:** ^1^ Centro de Investigación en Biotecnología Agroalimentaria (BITAL) Universidad de Almería Almería Spain; ^2^ Instituto de Biología Molecular y Celular de Plantas (UPV‐CSIC) Universidad Politécnica de Valencia Valencia Spain

**Keywords:** T‐DNA, enhancer trapping, *Solanum lycopersicum*, functional genomics, insertional mutagenesis, GUS expression

## Abstract

With the completion of genome sequencing projects, the next challenge is to close the gap between gene annotation and gene functional assignment. Genomic tools to identify gene functions are based on the analysis of phenotypic variations between a wild type and its mutant; hence, mutant collections are a valuable resource. In this sense, T‐DNA collections allow for an easy and straightforward identification of the tagged gene, serving as the basis of both forward and reverse genetic strategies. This study reports on the phenotypic and molecular characterization of an enhancer trap T‐DNA collection in tomato (*Solanum lycopersicum* L.), which has been produced by *Agrobacterium*‐mediated transformation using a binary vector bearing a minimal promoter fused to the *uidA* reporter gene. Two genes have been isolated from different T‐DNA mutants, one of these genes codes for a UTP‐glucose‐1‐phosphate uridylyltransferase involved in programmed cell death and leaf development, which means a novel gene function reported in tomato. Together, our results support that enhancer trapping is a powerful tool to identify novel genes and regulatory elements in tomato and that this T‐DNA mutant collection represents a highly valuable resource for functional analyses in this fleshy‐fruited model species.

## Introduction

Tomato (*Solanum lycopersicum* L.) is not only an important commercial crop because of its high nutritive value for both fresh market and processing industries, but it is also a model system for dicots, especially for fleshy fruit biology (Lozano *et al*., 2009; Meissner *et al*., 1997). Due to its numerous advantages, tomato is recognized as a representative Solanaceae species for agronomical and fundamental research (Gillaspy *et al*., 1993; Klee and Giovannoni, 2011; Ranjan *et al*., 2012; Tanksley, 2004). These advantages include its being easy to cultivate, short life cycle, high multiplication rate, self‐pollination and ease of mechanical crossing, together with a suitable transformation via *Agrobacterium tumefaciens* and the availability of its full genome sequence (The Tomato Genome Consortium, 2012).

Once the tomato genome sequence project has been completed, the challenge of the postgenome era is to determine the functions of the great number of genes annotated by the International Tomato Annotation Group (ITAG). The tomato nuclear genome has an estimated size of 950 Mb and consists of 12 chromosomes; its euchromatic portion contains ~220 Mb (Peterson *et al*., 1996), including more than 90% of the genes (Wang *et al*., 2006). Nevertheless, the majority of these genes have only been predicted by *in silico* analysis and their functions remain unknown or hypothetical (The Tomato Genome Consortium, 2009). Mutational analysis is one of the most efficient methods to isolate and understand gene functions. Thus, many spontaneous mutants have been preserved and characterized by the Tomato Genetic Resource Center (Chetelat, 2005). Furthermore, several chemical and physical mutagens have been used to generate new mutant populations (exhaustive data can be found on http://tgrc.ucdavis.edu/ and http://zamir.sgn.cornell.edu/mutants/). Nevertheless, the main disadvantage for both spontaneous and induced mutants is the difficulty to identify the mutated gene, which requires positional cloning and/or mapping‐by‐sequencing strategies (Schneeberger *et al*., 2009). Insertional mutagenesis using a transposon or T‐DNA insertion arises to solve this problem as the inserted element acts as a tag for gene identification. Although the potential of the maize *Ac/Ds* and *En/Spm* transposon systems has been demonstrated in different species such as Arabidopsis (Parinov *et al*., 1999; Raina *et al*., 2002; Speulman *et al*., 1999; Tissier *et al*., 1999), rice (Enoki *et al*., 1999; Greco *et al*., 2003) and tomato (Meissner *et al*., 2000), the T‐DNA insertional mutagenesis approach offers some advantages as T‐DNA integration is stable through generations and appears to be completely random (Tinland, 1996; Tzfira *et al*., 2004). In addition, the development of binary vectors has led to the generation of different T‐DNA insertional mutagenesis methods such as activation tagging (Memelink, 2003) or several ‘trapping’ systems like gene trapping, promoter trapping and enhancer trapping (Springer, 2000; Stanford *et al*., 2001). Thus, numerous T‐DNA mutant collections have been developed in Arabidopsis (Alonso *et al*., 2003; Campisi *et al*., 1999; Feldmann, 1991; Krysan *et al*., 1999; Qin *et al*., 2003; Sessions *et al*., 2002) and other crops like rice (Hsing *et al*., 2007; Jeon *et al*., 2000; Jeong *et al*., 2002; Wan *et al*., 2009; Wu *et al*., 2003). In tomato, two activation tagging collections have been generated in the cultivars Micro‐Tom (Mathews *et al*., 2003), a dwarf genotype bearing several mutations affecting plant development (Carvalho *et al*., 2011; Martí *et al*., 2006), and M82 (Carter *et al*., 2013), a processing tomato variety with determinate growth habit.

Enhancer trap system is a valuable tool for identifying regulatory elements. In the enhancer trap vectors, the reporter gene is fused to a minimal promoter, which is unable to drive the reporter gene expression alone but can be activated by neighbouring cis‐acting chromosomal enhancer elements (Springer, 2000; Stanford *et al*., 2001). Additionally, the enhancer trap system allows for the study of essential genes, as T‐DNA acts as a dominant element, whose expression pattern can be detected in hemizygous state (Campisi *et al*., 1999). Thus, enhancer trap lines could be selected by expression profiling and/or mutant phenotype. The enhancer trap system was first described in Drosophila (O'Kane and Gehring, 1987), and since then, it has been successfully used in several plant species such as Arabidopsis (Geisler *et al*., 2002; He *et al*., 2001; Sundaresan *et al*., 1995) and rice (Johnson *et al*., 2005; Peng *et al*., 2005; Sallaud *et al*., 2004; Wu *et al*., 2003). In this work, the previously described pD991 binary vector (Campisi *et al*., 1999) was used to produce more than 7800 enhancer trap lines, which make up the first tomato enhancer trap mutant collection. Furthermore, phenotypic and molecular characterization of transformed lines, as well as histochemical localization of β‐glucuronidase (GUS) activity in different plant tissues, proved the usefulness of enhancer trap mutagenesis as genomic tool for the identification of novel regulators of plant growth and reproductive development in tomato.

## Results

A large number of enhancer trap lines have been produced with the aim to develop an insertion‐based gene discovery system for tomato. The phenotypic characterization of these transgenic lines has made possible to identify mutants affected in plant growth and reproductive development. The main steps followed for the characterization of the enhancer trap mutant collection are described below (Figure S1) together with the genetic and molecular characterization of two T‐DNA mutants.

### Development of enhancer trap lines

The pD991 enhancer trap vector used in this work includes, at the 5′ end and close to the right border (RB), the *uidA* gene coding for GUS enzyme preceded by a minimal promoter, the latter being insufficient to drive GUS expression. In addition, the *NEOMYCIN PHOSPHOTRANSFERASE II* (*NPTII*) gene conferring kanamycin resistance is near the left border (LB) at the 3′ end of the T‐DNA (Figure S1a), and it is used as selection marker gene. Enhancer trap lines were generated from cocultured young leaf explants of tomato cultivars P73 and Moneymaker with the *Agrobacterium* strain LBA4404 carrying the binary vector pD991. Ploidy‐level analysis by flow cytometry showed that both diploid‐ and tetraploid‐independent transformants were generated; however, the percentage of diploid transgenic plants was higher in cv. Moneymaker (75.3%) than in cv. P73 (56.2%), despite the fact that transformation frequency was 32.6% and 43.2%, respectively (Table S1). For this reason, cv. Moneymaker was used as main genotype to increase the number of T‐DNA lines integrated in our functional genomic programme. Finally, a total of 7842 transgenic plants were generated, of which 5560 T0 lines were diploid, 1021 and 4539 T0 lines from P73 and Moneymaker tomato cultivars, respectively. Diploid T0 plants were then acclimated and subsequently grown under standard glasshouse conditions for further analysis so as to obtain their T1 progenies by selfing.

### Phenotypic screening of enhancer trap lines

**Figure 1:**
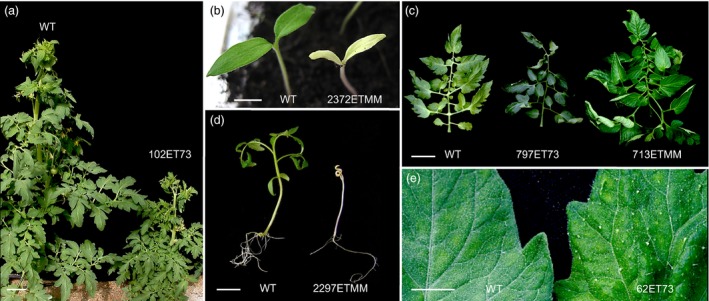
Representative phenotypes of enhancer trap lines altered in vegetative development. (a) Dwarf phenotype of the 102ET73 mutant. (b) 2372ETMM mutant showed chlorophyll deficiency in cotyledons. (c) Compared to wild type (left), T‐DNA mutants displayed dark green leaves, likely due to a high amount of chlorophyll (797ET73) and higher number of leaflets (713ETMM). (d) 2297ETMM mutant was defective in shoot apex growth and morphogenesis. (e) Leaves of the 62ET73 mutant showed higher density of trichomes. Scale bar = 10 cm in (a) and (c); and 1 cm in (b), (d) and (e).

**Figure 2:**
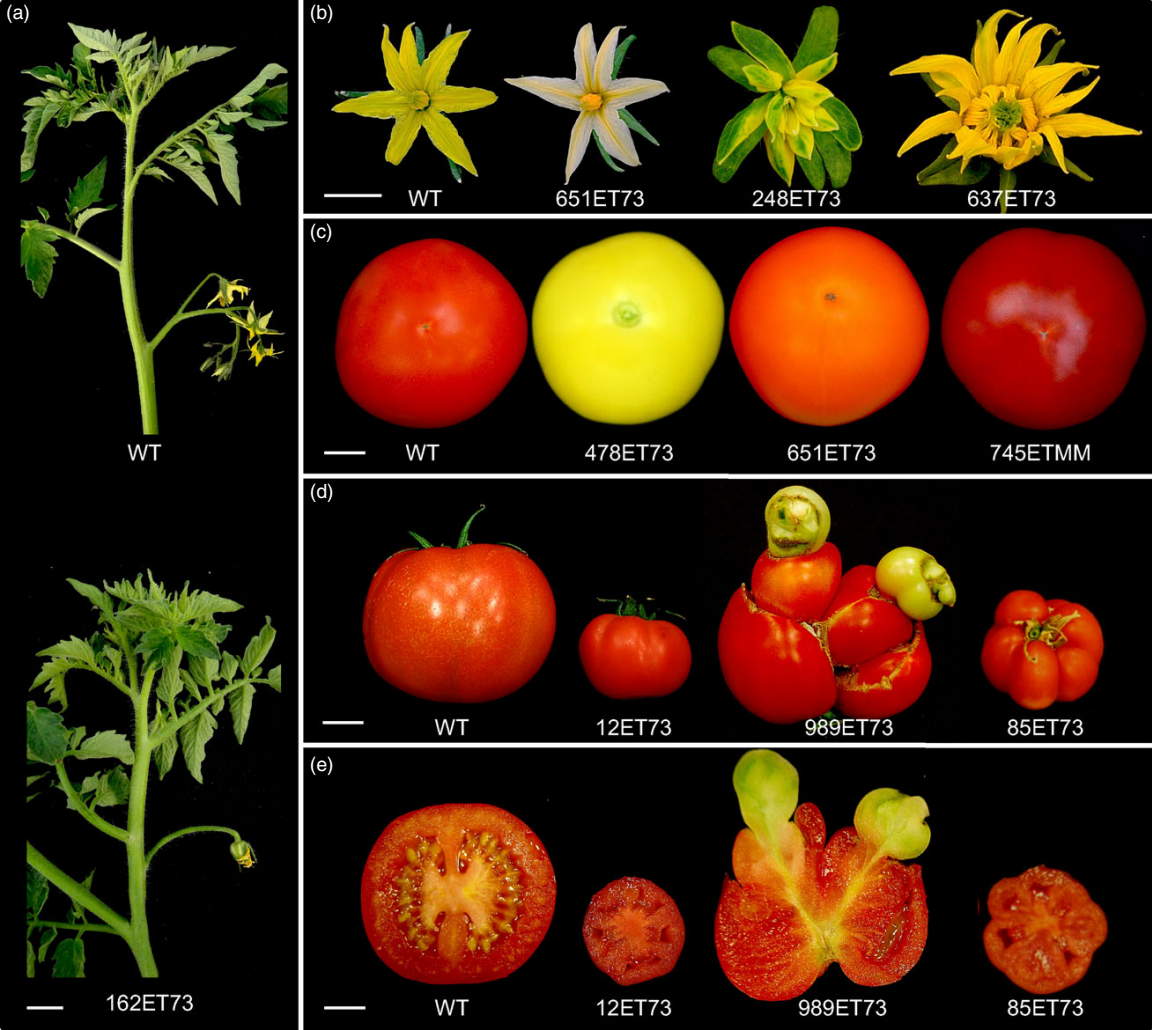
Representative phenotypes of enhancer trap lines affected in reproductive traits. (a) Inflorescences of wild‐type tomato plants were normally composed by 7–10 flowers (top), while the 162ET73 mutant line developed a single flower inflorescence (bottom). (b) From left to right, flowers from wild‐type and T‐DNA mutant lines showing alterations in the colour of petals and stamens (651ET73), homeotic conversions of floral whorls (248ET73) and an increased number of floral organs (637ET73). (c) From left to right, wild‐type fruit and T‐DNA mutant lines displaying yellow fruit (478ET73), orange fruit (651ET73) and intense red fruit (745ETMM). (d) From left to right, wild‐type fruit and fruits of three T‐DNA mutant lines (12ET73, 989ET73 and 85ET73) developing parthenocarpic (seedless) fruits with altered size and morphology. (e) Longitudinal sections of the same fruits showed in (d). Scale bar = 3 cm in (a); and 1 cm in (b), (c), (d) and (e).

A total of 4189 T1 transgenic plants were screened under glasshouse conditions (Figure S1b) to detect T‐DNA mutants affected in plant growth and reproductive development. Among them, 205 T0 lines displayed variations with respect to wild‐type (WT) untransformed plants. The inheritance pattern of the mutant phenotypes was confirmed by a T1 progeny analysis, which showed that the phenotype segregation fitted the expected ratio for a dominant mutation (3:1 for mutant and WT phenotypes) in most cases. In addition, 1858 T1 families were also characterized to identify recessive mutations. For this purpose, sixteen T1 plants from each family were cultivated under glasshouse conditions and screened for developmental alterations. Three hundred and seventeen of 1858 T1 families (17.1%) were found to display a mutant phenotype, and no differences in the relative frequency of mutants were found between Moneymaker and P73 cultivars. Mutant phenotypes observed in most T1 families (274 out of 317) segregated according to a monogenic recessive inheritance (3:1 for WT: mutant phenotypes), whereas 43 mutant lines showed complex inheritance patterns. Thus, it was found that enhancer trap lines displaying an altered vegetative development were affected in seedling development, shoot apex morphogenesis, plant size, leaf colour and morphology, and trichome density (Figure 1). Likewise, enhancer trap lines affected in reproductive traits were detected, such as flowering time, inflorescence architecture, flower colour and morphology, fruit pigmentation, fruit morphology and parthenocarpy (Figure 2). Among the phenotypic classes (Table 1), a high percentage of mutant lines were grouped in ‘plant size’ and ‘parthenocarpic fruit’ categories (31.2% and 21.1%, respectively), whereas the less frequent phenotype classes corresponded to flowering time (0.4%), flower abscission zone (1.2%) and cuticle/cracked fruit (1.2%).

**Table 1:** Catalogue of tomato mutant phenotypes

	Category	Dominants	Recessives	Complex inheritance^a^	Total	Frequency (%)
i.	Seedling lethality/albinism	1	16	0	17	3.3
ii.	Root development	2	19	1	22	4.2
iii.	Plant size	39	112	12	163	31.2
iv.	Leaf morphology and colour	27	25	1	53	10.2
v.	Senescence	5	19	3	27	5.2
vi.	Flowering time	1	1	0	2	0.4
vii.	Inflorescence architecture	9	13	0	22	4.2
viii.	Flower morphology and colour	6	10	2	18	3.5
ix.	Flower abscission zone	6	0	0	6	1.2
x.	Fruit set rate	26	8	0	34	6.5
xi.	Fruit morphology and colour	12	13	3	28	5.4
xii.	Seedless (parthenocarpic) fruit	61	31	18	110	21.1
xiii.	Ripening	5	6	3	14	2.7
xiv.	Cuticle/cracked fruit	5	1	0	6	1.2
	TOTAL	205	274	43	522	

a Complex inheritance: traits that do not follow strict Mendelian inheritance.

**Figure 3:**
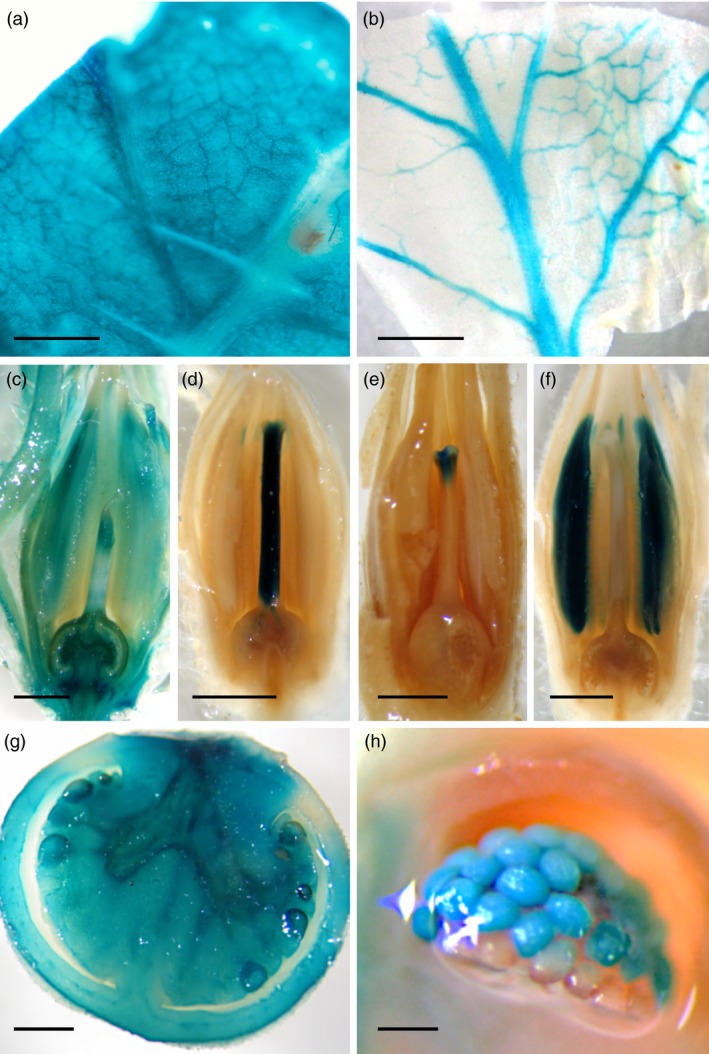
GUS expression patterns in enhancer trap lines. (a), (c) and (g) show organ‐specific GUS staining in leaf, flower and fruit, respectively. Tissue‐specific GUS expression was detected in vascular bundles of leaves (b), and in style (d), stigma (e), stamens (f) and ovules (h) of flowers. Scale bar = 1 cm in (a), (b) and (g); 0.25 cm from (c) to (f); and 50 μm in (h).

The GUS expression of *uidA* reporter gene was analysed in 836 T1 lines in order to provide a first overview about the organ and tissue expression specificity of genomic regions tagged by T‐DNA insertions (Figure 3). Results showed histochemical localization of GUS activity in vegetative and reproductive structures of almost all T0 lines (97.7%). Moreover, organ‐specific signals were exclusively found in vegetative organs (49 lines; Figure 3a,b), flowers (269 lines; Figure 3c‐f) or fruits (189 lines; Figure 3g,h). Interestingly, a significant number of mutant lines with organ‐specific GUS expression displayed a marked tissue or cell type specificity like that observed in vascular bundles of leaves (Figure 3b), and in several floral tissues (Figure 3d‐f).

### Characterization of T‐DNA integration sites

**Figure 4:**
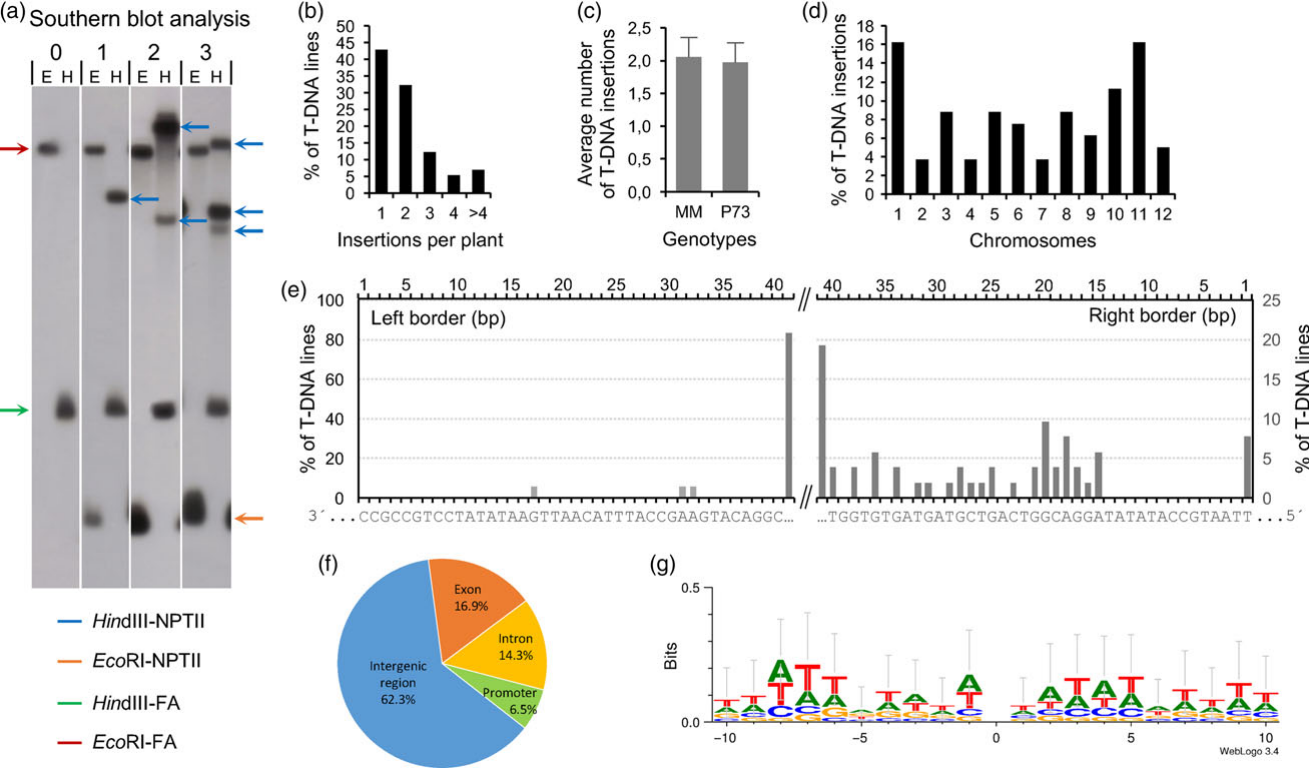
Molecular characterization of enhancer trap lines. (a) Southern blot analysis of genomic DNA digested by restriction enzymes *Eco* RI (e) and *Hin*dIII (h) and hybridized with the NPTII‐FA probe (for details, see Methods). (b) Number of T‐DNA insertions per T0 plant. (c) Average number of T‐DNA insertions in Moneymaker (MM) and P73 cultivars. (d) Distribution of T‐DNA insertions on tomato chromosomes. (e) Percentage of enhancer trap lines with deletions in the sequence of the integrated right (RB) and left (LB) borders (the last 40 bp are only shown). (f) Distribution of T‐DNA insertions on intergenic and genic regions. (g) WebLogo analysis of 20‐bp sequences surrounding the T‐DNA insertion sites. Zero position represents the insertion site.

The number of T‐DNA insertions in mutant lines was analysed by Southern blot hybridization using a chimeric probe composed by the *NPTII* (kanamycin resistance) and the tomato *FA* genes (Figures 4 and S1c). The hybridization generated *FA* fragments representing a positive control of the hybridization, that is a 10‐kb *Eco*RI‐FA fragment and a 1.9‐kb *Hin*dIII‐FA fragment, which were found in both transformed and WT plants (Figure 4a). In addition, a single 1‐kb *Eco*RI‐NPTII fragment was found in transformed plants, while the number of *Hin*dIII‐NPTII fragments indicated the number of T‐DNA insertions occurring in each line. Of 170 transgenic lines examined, 73 lines (42.9%) carried a single T‐DNA copy and the remaining had two or more T‐DNA copies (Figure 4b). The average number of T‐DNA insertions per T‐DNA line was 2.01 ± 0.9, and no significant differences were found in the average number of T‐DNA insertions between P73 and Moneymaker cultivars (t‐Student, *P *<* *0.05; Figure 4c).

DNA genomic fragments flanking the T‐DNA LB and RB sequences were identified by anchor PCR in 77 transgenic lines. After the sequencing of PCR products, sequence homology was firstly analysed using BLAST against the sequence of pD991 vector contained in the *A. tumefaciens* strain used for genetic transformation experiments. Results showed deletions of variable size affecting LB and RB sequences (Figure 4e). Indeed, LB was especially sensitive to T‐DNA integration as none of the T‐DNA lines analysed bore the complete LB sequence and almost all of them (99.8%) showed deletions larger than 40 bp. In addition, 15.6% of mutants were found to have aberrant T‐DNA insertions, which were due to rearrangements either within T‐DNA fragment or involving vector backbone. Secondly, once the vector sequences were removed from the amplified flanking region, the homology search of the trimmed sequences was carried out using BLAST against the tomato genome database (http://solgenomics.net). Results showed that T‐DNA insertions were distributed over all chromosomes; however, a bias towards chromosomes 1 and 11 was detected despite that no correlations with the euchromatin ratio or gene content of these chromosomes were found (Figure 4d and S2).

The genomic sequences flanking the T‐DNA were analysed to further characterize the chromosome regions where the T‐DNA was inserted in the tomato genome. Thus, 37.7% of insertions were located in either the coding or the promoter region of annotated genes, which was arbitrarily defined up to 2 kb upstream from the transcription start codon. Among them, 16.9% and 14.3% were positioned in exons and introns, respectively, while 6.5% were found in promoter regions. The remaining 62.3% of T‐DNA insertions were placed in intergenic regions (Figure 4f). Furthermore, most of T‐DNA insertions were found to be located in euchromatic DNA (75.4%, Figure S2).

The nucleotide composition of the sequences surrounding the insertion sites (SSIS) was also ascertained to determine whether there was a preference for insertions in particular regions. The analysis of 100‐bp sequences, 50 bp upstream and downstream of each insertion site, displayed a GC content of 34.9% in the SSIS. An additional analysis performed with WebLogo software using 20‐bp SSIS revealed a nonconsensus sequence in the T‐DNA integration site, as well as a rich AT content (65.6%) in both RB and LB flanking sequences (Figure 4g). As expected, the genes tagged by T‐DNA encoded a wide variety of proteins such as transcription factors, plant metabolism enzymes or membrane receptors. Examples of T‐DNA locations and other relevant information about tagged flanking sequences are shown in Table 2.

**Table 2:** Examples of insertion sites of enhancer trap T‐DNAs in the tomato genome

Line	RB/LB^a^	Ch.^b^	Region	Gene	Protein function
746ET73	RB	1	Intron	*Solyc01g010500*	Ein3‐binding f‐box protein 3
374ETMM	RB	1	Exon	*Solyc01g095030*	MYB Transcription factor
282ET73	LB	3	Exon	*Solyc03g005580*	Legumin 11S‐globulin
1381ETMM	RB	5	Exon	*Solyc05g009390*	Lipase‐like protein
515ETMM	RB	5	Promoter	*Solyc05g012020*	MADS‐box transcription factor
386ETMM	RB	5	Exon	*Solyc05g013480*	ATP‐dependent protease
136ETMM	RB	5	Exon	*Solyc05g013530*	Octicosapeptide
832ETMM	RB	6	Promoter	*Solyc06g008020*	Zinc Finger Transcription factor
1336ETMM	RB	6	Promoter	*Solyc06g068090*	Phospholipase PLDa1
390ETMM	RB	6	Exon	*Solyc06g068980*	Serine/threonine‐protein kinase B‐raf
1635ETMM	LB	8	Intron	*Solyc08g007380*	Histidine triad protein
365ET73	RB	8	Exon	*Solyc08g061240*	Catalytic/hydrolase
51ET73	RB	10	Exon	*Solyc10g049460*	Transposon Ty1‐A Gag‐Pol polyprotein
740ET73	RB	10	Intron	*Solyc10g083250*	RNA‐binding protein
1527ETMM	RB	11	Intron	*Solyc11g008620*	Phosphoglycolate phosphatase
2477ETMM	RB	11	Exon	*Solyc11g011960*	UTP‐glucose‐1‐phosphate uridylyltransferase
651ET73	RB	11	Exon	*Solyc11g069740*	Nitrate transporter

a T‐DNA flanking genomic sequences were amplified from RB: right border or LB: left border.

b Ch: Chromosome.

### Molecular isolation of two T‐DNA tagged mutants: proof of concept

**Figure 5:**
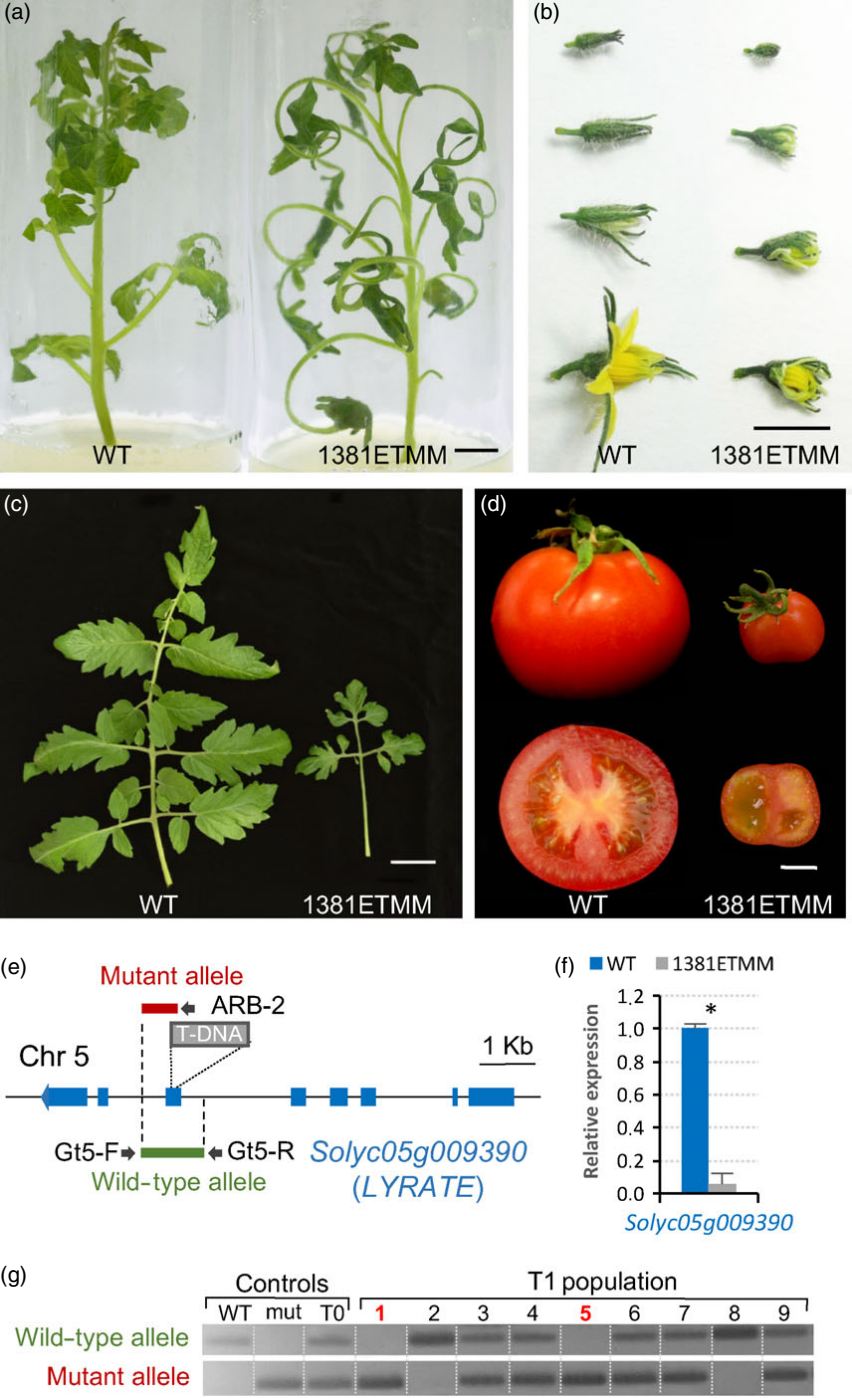
Phenotypic and molecular characterization of the 1381ETMM line. Mutant plants of the 1381ETMM line were affected in the development of leaves (a, c), flowers (b) and fruits (d). (e) Schematic representation of T‐DNA localization in the 1381ETMM line. (f) Relative expression of *LYRATE* (*Solyc05g009390*) in wild‐type and 1381ETMM mutant plants. Asterisk denotes significant differences at *P *<* *0.05. (g) Cosegregation analysis of T‐DNA insertion and 1381ETMM mutant phenotype. Red numbers indicate plants displaying mutant phenotype. Scale bar = 1 cm in (a), (b) and (d); and 5 cm in (c).

As proof of concept, here we describe the molecular characterization of two selected T‐DNA mutant lines named 1381ETMM and 2477ETMM. The segregation ratio observed in the T1 progeny of the line 1381ETMM was consistent with a monogenic recessive inheritance for the mutant phenotype (16 WT: 8 mut; χ^2^ = 0.89, *P *=* *0.35), which is characterized by a significant reduction in leaf size, giving rise to only one or two secondary leaflets (Figure 5a,c). In addition, flower development was severely altered as mutant plants produced flowers with reduced petals that opened prematurely (Figure 5b). These flowers rarely yielded fruits, and when they did, fruits were parthenocarpic (seedless) and smaller compared with WT fruits (Figure 5d). Southern blot analysis showed that 1381ETMM line only bore a single T‐DNA copy. Cloning of T‐DNA flanking genomic sequences revealed that T‐DNA was inserted at position 3 537 861 on chromosome 5 (ITAG2.4), in the sixth exon of the *LYRATE* gene (*Solyc05g009390*), which codes for a lipase‐like protein (Figure 5e) involved in leaf development (David‐Schwartz *et al*., 2009). The effects of T‐DNA integration on gene expression were determined by quantitative RT‐PCR, which showed that *LYRATE* was significantly down‐regulated in 1381ETMM mutant tissues compared with WT (Figure 5f).

**Figure 6:**
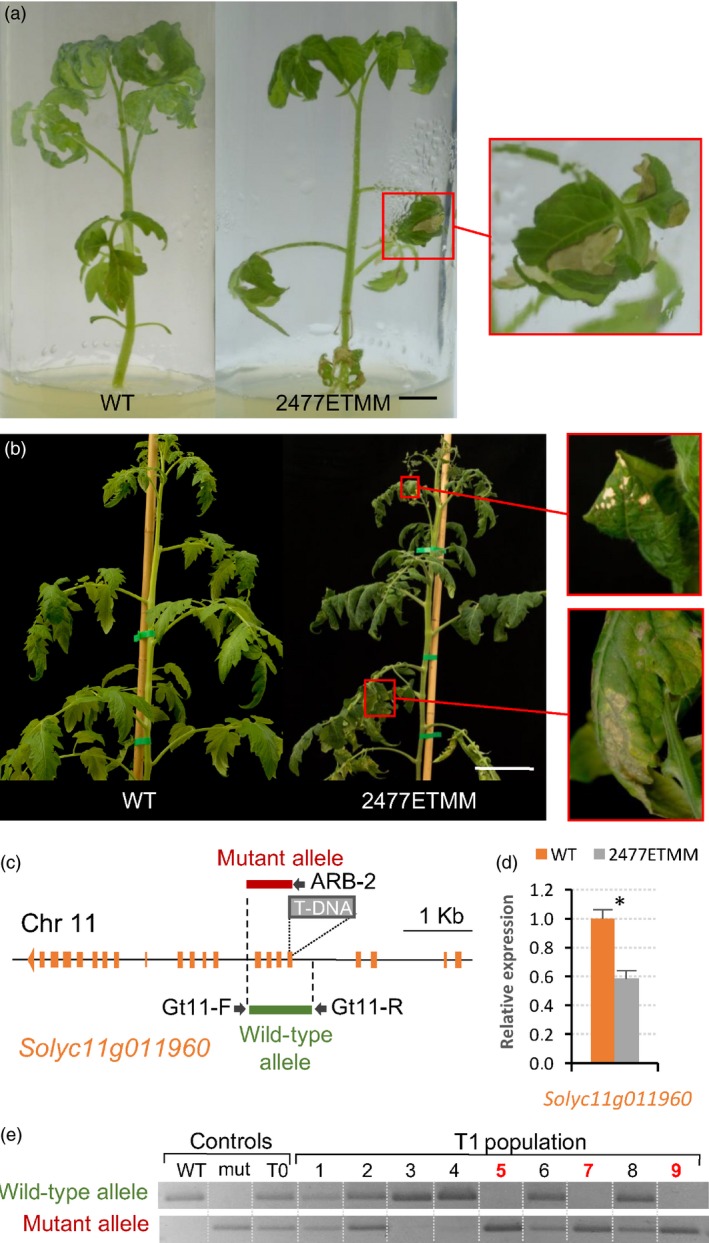
Phenotypic and molecular characterization of the 2477ETMM line. Necrosis of leaf tissues observed in the mutant phenotype of 2477ETMM line when plants grew either under *in vitro* (a) or glasshouse (b) conditions (magnification pictures of necrotic tissues are shown in right panels). (c) Schematic representation of T‐DNA integration site in the 2477ETMM line. (d) Relative expression of the gene coding the UTP‐glucose‐1‐phosphate uridylyltransferase (*Solyc11g011960*) in wild‐type and 2477ETMM mutant genotypes. Asterisk denotes significant differences at *P *<* *0.05. (e) Cosegregation analysis of T‐DNA insertion and 2477ETMM mutant phenotype. Red numbers indicate plants displaying mutant phenotype. Scale bar = 1 cm in (a) and 5 cm in (b).

Regarding the line 2477ETMM, a segregating population of 20 plants was evaluated, which segregated according to a monogenic recessive inheritance for the mutant phenotype (14 WT: 6 mut; χ^2^ = 0.27, *P *=* *0.61). At early stages of development, leaves of mutant plants showed evident necrosis symptoms, which affected all leaf tissues, and led to a loss of photosynthetic tissue and a reduction in plant growth (Figure 6a,b). This mutant phenotype was observed in young plants developed under both *in vitro* and glasshouse conditions. Later in the development, necrosis increased and the affected leaves became curled and senescent (Figure 6b). Southern blot experiments displayed a single T‐DNA insertion in the mutant plants of 2477ETMM line, and the analysis of T‐DNA flanking sequences revealed that T‐DNA was integrated at position 4 916 541 on chromosome 11 (ITAG2.4), in the fifth exon of the *Solyc11g011960*, a gene coding for a UTP‐glucose‐1‐phosphate uridylyltransferase (Figure 6c). Expression analysis showed that T‐DNA integration led to a decreased level of transcripts of the tagged gene in 2477ETMM mutant tissues (Figure 6d).

With the aim to support the insertional nature of the mutant phenotypes above described, a cosegregation analysis of the T‐DNA insertion with the mutant phenotype was assessed in T1 segregating populations (Figures 5g and 6e). In both cases, all mutant plants bore T‐DNA insertion in the homozygous state, whereas WT plants were azygous or heterozygous for T‐DNA, which suggested that mutant phenotypes were caused by the T‐DNA insertion occurring in each line. Nevertheless, the evaluation of 77 selected T‐DNA lines showed no cosegregation between the mutant phenotype and the T‐DNA insertion in 26 of these lines (33.8%). These results suggested that somaclonal variation events, partial T‐DNA or vector backbone fragment insertions and chromosomal rearrangements may have occurred during the *in vitro* genetic transformation, similarly to that reported in other model plant species (Feldmann, 1991; Miyao *et al*., 2007).

In addition, different strategies were developed to further confirm that the tagged genes in the 1381ETMM and 2477ETMM lines were responsible for the mutant phenotypes observed. For the 1381ETMM line, a complementation test was carried out by crossing wild‐type heterozygous plants, one bearing the 1381ETMM mutation (female parent) and the other carrying the *lyrate* mutation (*lyr2*, accession number LA2923, male parent), as homozygous mutant plants for each mutant allele rarely developed fertile flowers. The evaluation of the F1 offspring (Figure S3) showed the expected 3:1 segregation of wild‐type and mutant phenotypes (18 WT: 8 mut; χ^2^ = 0.50, *P *=* *0.46), which confirmed that the 1381ETMM mutation is a new allele of the *LYRATE* gene. Regarding the 2477ETMM line, a RNA interference (RNAi) strategy was carried out to silence the expression of the *Solyc11g011960* tagged gene (Figure S4). Thus, 10 independent transformants were obtained and used for phenotypic characterization, three of which were selected by their diploid nature and their reduced expression levels (less than 0.1‐fold change relative to WT plants). These T0 RNAi lines displayed a similar mutant phenotype of that reported for the 2477ETMM line, particularly leaves with evident necrosis symptoms and a reduction of plant growth (Figure S4). These results supported that the T‐DNA insertion located at the *Solyc11g011960* gene is responsible for the mutant phenotype observed in the 2477ETMM line.

## Discussion

Considerable progress has been made in developing genomic resources for tomato, including the release of the complete genome sequence (The Tomato Genome Consortium, 2012). As a result, 34 727 protein‐coding genes were annotated by the ITAG consortium, most of them with unknown functions. Therefore, a key research priority is to develop a set of tools to assign functions to the predicted gene sequences, thus facilitating that this genomic information can be applied in tomato genomics‐assisted breeding. Insertional mutagenesis is one of the most suitable and direct approaches to define gene functions. A tomato activation tagging insertional mutant collection was developed by Mathews *et al*. (2003) using Micro‐Tom, a miniature variety originally bred for ornamental purposes (Scott and Harbaugh, 1989). The dwarf phenotype of Micro‐Tom plants is determined by a combination of hormonal and photomorphogenetic mutations (altered sensitivity or endogenous levels of auxin, ethylene, abscisic acid, gibberellin, brassinosteroid and light response) into its genetic background (Carvalho *et al*., 2011; Martí *et al*., 2006), which may make this genotype unsuitable for the identification of genetic factors controlling important developmental traits like those related to plant vigour and fruit size. In this study, two commercial tomato cultivars, that is Moneymaker and P73, with adequate agronomic performance have been used to develop a large‐scale insertional mutagenesis approach. Thus, more than 5500 diploid T0 lines have been generated using the *Agrobacterium*‐mediated transformation protocol with the pD991 enhancer trap vector. The average transformation frequency was 32.4% in cv. Moneymaker and 42.9% in cv. P73; in both cases, it was higher than previously described by Hu and Phillips (2001) for the industrial processing cultivar UC82 (25%), although lower than previously reported by Dan *et al*. (2006) for the Micro‐Tom variety (57%). Both this work and the two previously mentioned reports used an *Agrobacterium tumefaciens*‐mediated transformation procedure, which corroborates that T‐DNA integration into tomato genomes highly depends on the genotype (Ellul *et al*., 2003).

The *in vivo* screening of an insertional mutant collection is a space‐ and time‐consuming process, particularly in tomato cultivars like Moneymaker and P73, which show an indeterminate growth habit and have 4‐ to 6‐month‐long life cycles, as it is characteristic of tomato varieties for fresh consumption market. Thus, for more than 6 years (two seasons per year, i.e. autumn–winter and spring–summer), a total of 4189 T0 plants and 1858 T1 progenies (16 plants of each T1 line) were evaluated under glasshouse conditions. Based on this evaluation, mutant lines with defective vegetative (Figure 1) and reproductive (Figure 2) development were found; most of them belonged to ‘plant size’ (31.2%) and ‘parthenocarpic fruit’ (21.1%) categories (Table 1). Two hundred and five of the 522 mutant lines identified had an autosomal dominant mode of inheritance, which means that a novel dominant mutated allele was generated in 4.9% of the evaluated T0 lines. This percentage was smaller than that previously reported by Mathews *et al*. (2003) in the Micro‐Tom activation tagging collection, where 12.8% of T0 lines evaluated showed a dominant mutant phenotype. Such differences might be due to the transformation method or the tomato genetic background used in each study. However, it should be taken into account that Mathews *et al*. (2003) characterized a small number of T1 progenies to confirm the mutant phenotypes observed in T0 selected plants; hence, it is not possible to compare the percentage of recessive mutant lines detected in both T‐DNA collections. On the other hand, the present study evaluated a limited number of discrete traits; hence, if enhancer trap lines were screened under other conditions such as drought or temperature stress conditions or under pathogen pressure, phenotypic description of this collection would be much more enriched, which would allow for the identification of new mutant phenotypes. In fact, the same pD991 vector‐based gene construct has been used to generate a T‐DNA mutant collection in the wild‐related species *S. pennellii* whose screening has provided useful information regarding regulatory genes involved in salt stress response (Atarés *et al*., 2011).

In addition, the expression of the reporter *uidA* gene was evaluated in vegetative structures, flowers and immature fruits. While different GUS patterns were detected in the enhancer trap lines (Figure 3 and Table S2), a significant percentage of these lines (60.6%) displayed organ‐ or tissue‐specific GUS activity. The combined use of T‐DNA‐based mutagenesis and GUS histochemical detection has also been carried out in other Solanaceae species, such as *Nicotiana tabacum* and *S. tuberosum* (Goldsbrough and Bevan, 1991; Lindsey *et al*., 1993; Topping *et al*., 1991). Using the weak (−90 bp) CaMV35S promoter, Topping *et al*. (1991) analysed patterns of GUS gene expression in a collection of 184 tobacco T‐DNA lines, from which 73% displayed GUS activity with different organ and tissue specificities. Comparable results were observed by Goldsbrough and Bevan (1991) in potato T‐DNA lines, using a similar enhancer trap vector. Thus, different patterns of GUS expression were detected at high frequency. Likewise, similar findings were reported by Lindsey *et al*. (1993) in T‐DNA lines of tobacco, tomato and Arabidopsis. The percentage of lines showing GUS activity was high for all three species; however, the frequencies of GUS activity detected in a given organ were different among species, which ranged from 25% in stems of potato and 30% in roots of Arabidopsis, up to 92% in flowers of tobacco T‐DNA lines. Therefore, the set of T‐DNA lines here reported showing specific GUS expression in the flower and fruit tissues (269 and 189 lines, respectively) could be used to further studies of functional genomics in tomato. Among them, the high percentage of lines (26.2%) displaying a stamen‐specific GUS staining pattern is remarkable. As defects during pollen ontogeny produce parthenocarpic (seedless) fruits, such percentage is in agreement with that of mutant lines with parthenocarpic fruit (21.1%) identified during phenotypic screening under glasshouse conditions. In fleshy fruit plants like tomato, parthenocarpy is considered to be of commercial importance as seedless fruits usually have increased fruit quality traits, and parthenocarpic varieties can provide tomato yield under unfavourable climatic conditions (Gorguet *et al*., 2005; Pandolfini, 2009). Therefore, these mutant lines could help to uncover novel genes, which may exert a fundamental role during pollen and fruit developmental processes. Moreover, enhancer trapping is suitable for isolating regulatory genes involved in developmental traits which may be difficult to address from mutants showing highly pleiotropic or lethal phenotypes. In this case, gene discovery mostly depends on the reporter gene expression rather than the mutant phenotype (Groover *et al*., 2004).

As regards molecular characterization, Southern blot analysis revealed that enhancer trap lines contain an average of 2.01 T‐DNA insertions although 43% of assessed lines bear a single T‐DNA copy. This result is in accordance with that reported by Wu *et al*. (2003) in a rice enhancer trap collection; however, it differed from the 1.4 T‐DNA insertions found as average in Arabidopsis and rice mutant collections developed by other trapping systems (Feldmann, 1991; Jeon *et al*., 2000). Examination of the junctions between the T‐DNA borders and tomato genomic DNA revealed that right and left borders were not completely integrated (Figure 4e). The deletions in the left border junction were even more severe (deletions larger than 40 bp). Nevertheless, this phenomenon seems to be common in *Agrobacterium*‐mediated T‐DNA‐transferring processes as it had also been previously found in Arabidopsis and rice (Hiei *et al*., 1994; Tinland, 1996; Wu *et al*., 2003). The rationale of this phenomenon is that T‐DNA integration into plant genome is usually achieved by a form of illegitimate recombination, which is initiated by a break in the DNA involved in the mutational process (Gheysen *et al*., 1991). Recombination of only a few identical nucleotides preferentially occurs at the base where VirD2 protein nicks the right border, as T‐DNA transfer is a polar process, which is initiated at the right border and ends at the left border. However, the left junction between bacterial and plant DNA frequently does not occur within the left border sequence, which results in the commonly found deletion of left border sequences (Rossi *et al*., 1996; Tinland *et al*., 1994, 1995).

In the present study, 75.4% of T‐DNA insertion occurred in these large gene‐rich euchromatic regions, where 62.3% were located in intergenic regions (Figure 4f). This result further supports the significant percentage of enhancer trap lines showing GUS activity and agrees with previous studies which have reported that T‐DNA integration favours intergenic regions over genic regions (Alonso *et al*., 2003; Krysan *et al*., 1999; Pan *et al*., 2005; Rosso *et al*., 2003). In the Solanaceae family, fluorescence *in situ* hybridization (FISH) experiments carried out in Petunia indicated that T‐DNA insertions occur preferentially in distal chromosome regions, where gene density is higher and chromatin is loosely packed and transcriptionally active (Ten Hoopen *et al*., 1996; Wang *et al*., 1995). Likewise, comparative analysis in Arabidopsis and rice revealed that T‐DNA insertions were randomly found in the Arabidopsis genome, which contains little repetitive DNA and is globally rich in gene concentration whereas in the rice genome, T‐DNA fragments were inserted in gene‐dense euchromatic regions (Barakat *et al*., 2000; Zhang *et al*., 2007). Furthermore, GC content (34.9%) in sequences surrounding the T‐DNA insertion sites was similar to that previously reported by Barakat *et al*. (2000) and Qin *et al*. (2003) in Arabidopsis and rice insertional collections, suggesting that T‐DNA integration events most likely occur in genome sequences having a moderate GC content.

As proof of concept, we have reported the isolation of the genes tagged in two T‐DNA lines. Firstly, a new T‐DNA allele of the *LYRATE* gene has been identified from the 1381ETMM line. *LYRATE* was found to be the tomato homologue of the *Arabidopsis JAGGED* gene, and the functional analysis proved that it functions as crucial regulator of leaf development and patterning by interacting with other transcriptional factors (David‐Schwartz *et al*., 2009). Mutations at the *LYRATE* locus also affected the proper development of floral organs, mainly stamens and carpels, as well as fruit formation, which were likely due to pleiotropic effects. Our results corroborated the functional analysis of *LYRATE* and provided a new allele for further insight into the molecular and physiological mechanisms underlying complex biological processes such as vegetative and reproductive development. In addition, the gene coding for a UTP‐glucose‐1‐phosphate uridylyltransferase, an enzyme involved in the biosynthesis of carbohydrate cell components, such as cellulose and callose, was isolated from the 2477ETMM line. Phenotypic characterization of the T‐DNA mutant suggests that this gene should play an important role in regulating cell death during leaf development of tomato. In *A. thaliana,* an *UTP‐GLUCOSE‐1‐PHOSPHATE URIDYLYLTRANSFERASE* homologue (*UGP1*) gene has been reported as a crucial regulator of programmed cell death (Chivasa *et al*., 2013), which supports our hypothesis on the role of the tagged gene. Furthermore, *UGP1* and *UGP2* seem to act redundantly in plant growth and reproduction in *A. thaliana* (Park *et al*., 2010), suggesting that UTP‐glucose‐1‐phosphate uridylyltransferase may have overall housekeeping functions during plant development. Noteworthy, our results in tomato also provide a suitable scenario for further functional and evolutionary studies on the *UTP‐GLUCOSE‐1‐PHOSPHATE URIDYLYLTRANSFERASE* genes. Likewise, the screening of this T‐DNA mutant collection has allowed us to identify other tagged mutants. Among them are *vegetative inflorescence* (*mc‐vin*) and *altered response to salt stress 1* (*ars1*) mutants (Campos *et al*., 2016; Yuste‐Lisbona *et al*., 2016). All together, these results strongly support the usefulness of enhancer trapping as an efficient strategy for functional genomics, allowing for the discovery of novel genes and regulatory elements.

## Experimental procedures

### Generation of enhancer trap lines

The enhancer trap vector used for transformation was pD991 (kindly supplied by Dr. Thomas Jack; Department of Biological Sciences, Dartmouth College, USA), which was described by Campisi *et al*. (1999). Young leaf explants were transformed with *A*. *tumefaciens* strain LBA4404 following the protocol described by Ellul *et al*. (2003). The transformed plants (T0) were selected by growing the explants in the salt medium reported by Murashige and Skoog (1962), sucrose (10 g/L) and kanamycin (100 mg/L). To ensure that each regenerated plant represented an independent transgenic event, only one regenerated plant from a single poked area of an inoculated leaf explant was selected. Transformation frequency was estimated as the number of independent transgenic events divided by the total number of inoculated leaf explants and then multiplied by 100. Furthermore, the ploidy level in transgenic plants was evaluated according to the protocol described by Atarés *et al*. (2011). Thus, the diploid plants from the T‐DNA insertion lines were selected and labelled with a consecutive number and the tag ‘ET73’ or ‘ETMM’, depending on whether the callus was originated from P73 or Moneymaker cultivars, respectively. Seeds of Moneymaker (accession LA2706) were obtained from Tomato Genetics Resource Center (TGRC, http://tgrc.ucdavis.edu/), whereas P73 seeds were kindly provided by Dr. M.J. Díez (COMAV‐UPV, Valencia, Spain). Several clonal replicates for each T0 line were obtained by culturing axillary buds in rooting medium. These replicates were used to maintain the T‐DNA collection under *in vitro* growth conditions as well as to acclimatize a sufficient number of replicates under glasshouse conditions to identify dominant insertion mutants and obtain T1 seeds by selfing. The collected T1 seeds were dried and catalogued in a temperature‐ and humidity‐controlled chamber. Furthermore, in order to detect recessive insertion mutants, sixteen T1 plants from each progeny were cultivated under glasshouse conditions for two seasons each year (autumn–winter and spring–summer) from 2009 to 2015.

### Phenotypic characterization

Vegetative and reproductive relevant traits were considered for phenotypic characterization (depicted in Table 1). Consequently, the mutant lines were classified into 14 phenotypic categories according to criteria described by Menda *et al*. (2004) with several modifications: (i) seedling lethality/albinism, mutations affecting embryo survival and absence or deficiency of chlorophyll during seedling; (ii) root development, that is altered root morphogenesis; (iii) plant size, from the soil surface to the apex at the fifteenth leaf stage; (iv) leaf morphology and colour, reflected by alterations in size, colour and complexity of leaf and leaflet (margin, venation, shape), as well as an increase or decrease in the number of trichomes; (v) senescence, that is premature death of the plant; (vi) flowering time, measured as the number of leaves before flowering; (vii) inflorescence architecture, comprised by variations in the number of inflorescences and the number of flowers per inflorescence; (viii) flower morphology and colour, including any mutants with homeotic changes, as well as alterations in size and colour; (ix) flower abscission zone, mutations affecting abscission layer development that cause alteration in flower dropping; (x) fruit set rate, measured as the proportion of flowers that yielded fruits compared with the wild type; (xi) fruit morphology and colour, reflected by variations in size and shape (rounded, elliptic, heart shape, among others) and colour (variation not due to late ripening, e.g. orange, yellow, green); (xii) seedless (parthenocarpic) fruit, comprising those mutants with any case of partial or full sterility which gave rise to parthenocarpy (absence of seeds) or stenospermocarpy (contain only rudiments of seeds) fruits; (xiii) fruit ripening, measured as fruit firmness compared with the wild type; and (xiv) cuticle/cracked fruit, mutations affecting fruit cuticle, epidermis and pericarp properties. Measurements were taken in centimetres and weight in grams.

### GUS assay

A histochemical GUS assay was conducted as described by Atarés *et al*. (2011). Different tissues of T0 transformed plants were placed in GUS staining solution [100 mm sodium phosphate at pH 7.0, 10 mm ethylenediaminetetraacetic acid (EDTA), 0.1% Triton X‐100, 0.5 mg/mL X‐Gluc, 0.5 mm potassium ferricyanide, 0.5 mm potassium ferrocyanide and 20% methanol] and incubated at 37 °C for 20–24 h. Subsequently, the GUS‐stained tissues were washed with 70% ethanol and examined under a zoom stereomicroscope (MZFLIII, Leica). Three replicates of each sample were analysed.

### DNA isolation

Tomato genomic DNA was isolated according to Dellaporta *et al*. (1983). Genomic DNA was quantified by fluorometry using SYBR Green I (Sigma‐Aldrich) as fluorophore. Fluorescence measurements were made at room temperature using Synergy MX (Biotek) fluorometer.

### Southern blot analysis

The number of T‐DNA insertions existing in selected mutants was determined by Southern blot. DNA blot hybridization was performed as described by Ausubel *et al*. (1995) using 10 μg of genomic DNA digested by restriction enzymes *Eco*RI and *Hin*dIII, electrophoresed throughout 0.8% agarose gel in 1X TBE buffer (100 mm Tris‐borate, 1 mm EDTA, pH 8.3), and blotted onto Hybond N+ membranes (GE Healthcare). Hybridization was carried out with a chimeric probe, fusing the complete coding sequence of the *NPTII* gene to 811 bp of coding sequence of endogenous tomato *FALSIFLORA* (*FA*) gene, which was employed as hybridization positive control. Finally, the chimeric *FA*‐*NPTII* probe (1635 bp) was labelled with [α‐32P]dCTP using High Prime random priming kit (Roche Applied Science) following the manufacturer's instructions. Nylon membranes were exposed to Hyperfilms (GE Healthcare).

### Identification of T‐DNA flanking sequences

The T‐DNA flanking sequences were isolated by anchor PCR according to the protocol previously established by Schupp *et al*. (1999) and Spertini *et al*. (1999) with some modifications: (i) genomic DNA (500 ng) was digested with blunt‐end restriction enzymes *Eco*RV, *Dra*I, *Sca*I, *Stu*I, *Alu*I, *Hin*cII, *Pvu*II or *Sma*I; (ii) additional third nested PCR was employed to avoid nonspecific amplification products; and (iii) new specific primers were designed for the RB, LB and Adapter. The sequence of primers used is listed in Table S3. PCR products were sequenced using the BigDye Terminator Cycle Sequencing Ready Reaction Kit (Applied Biosystems) following the manufacturer's instructions. The cloned sequences were compared with SGN Database (http://solgenomics.net/tools/blast/) to assign the T‐DNA insertion site on tomato genome. Furthermore, flanking sequences tags were examined to search for an integration pattern sequence using the WebLogo v3.4 software (http://weblogo.threeplusone.com/) described in Crooks *et al*. (2004).

### PCR genotyping

Cosegregation of the T‐DNA insertion site with the mutant phenotype in the T1 progeny for selected mutants was checked by PCR using (i) the specific genomic forward and reverse primers to amplify the wild‐type allele (without T‐DNA insertion) and (ii) one specific genomic primer and the specific T‐DNA border primer (from RB or LB) to amplify the mutant allele (carrying the T‐DNA insertion). The primers located upstream and downstream of the T‐DNA insertional sites in each line were designed based on sequence information available from SGN Database (http://solgenomics.net/). The sequence of genotyping primers used is listed in Table S3.

### Tomato RNA isolation and qRT‐PCR analysis

Total RNA was isolated using TRIzol (Invitrogen) following the manufacturer's instructions from young leaves. The DNA‐freeTM kit (Ambion) was used to remove contaminating DNA from each sample. The cDNA was synthetized by M‐MuLV reverse transcriptase (Fermentas Life Sciences) with a mixture of random hexamer and oligo(dT)_18_ primers. Specific primer pairs for each evaluated gene were described in Supplementary Table S3. Gene expression analysis was performed with three biological and two technical replicates using SYBR Green PCR Master Mix (Applied Biosystems) kit and the 7300 Real‐Time PCR System (Applied Biosystems). The ∆∆Ct calculation method (Winer *et al*., 1999) was used to express the results in arbitrary units by comparison with a data point from the wild‐type samples. The housekeeping gene *Ubiquitine3* (*Solyc01g056940*) was used as a control. Means of WT and mutant samples were compared using a least significant difference (LSD) test (*P *<* *0.05).

### Generation of silencing lines

A RNA interference (RNAi) approach was followed to generate *Solyc11g011960* silencing lines. A 117‐bp fragment of the *Solyc11g011960* cDNA was amplified using the primers RNAi‐F (5′‐TCTAGACTCGAGGGTTTGGATCTAGCGTTACCC‐3′) and RNAi‐R (5′‐ATCGATGGTACCCCTCAGGTCCATTGATGTCC‐3′), and the PCR product was cloned in sense and antisense orientation separated by intronic sequences into the pKannibal vector, which was then digested with *Not*I and cloned into the binary vector pART27, according to Campos *et al*. (2016). The binary plasmids generated were transformed with *A*. *tumefaciens* strain LBA4404 following the protocol described by Ellul *et al*. (2003).

## Conflict of interest

The author(s) declare that they have no competing interests.

## Supporting information


**Figure S1** Schematic representation of the T‐DNA insertional mutagenesis programme described in this work.
**Figure S2** Graphical representation of the distribution of T‐DNA insertions on tomato chromosomes.
**Figure S3** Complementation test of 1381ETMM and *lyrate* mutations.
**Figure S4** Phenotypic characterization of RNA interference (RNAi) lines for the *Solyc11g011960*, which was the gene tagged by the T‐DNA insertion in the 2477ETMM line.
**Table S1** Transformation efficiency in two tomato cultivars.
**Table S2** Summary of reporter GUS expression.
**Table S3** Primer sequences used for anchor PCR, genotyping and qRT‐PCR analyses.
