# Supplementary material for: A collection of enhancer trap insertional mutants for functional genomics in tomato
Source: Plant Biotechnol J. 2017 Apr 20;15(11):1439–52. doi: 10.1111/pbi.12728 (PMC5633825; doi:10.1111/pbi.12728)
Supplement: Supplementary file 1 — Figure S1 Schematic representation of the T‐DNA insertional mutagenesis programme described in this work. Figure S2 Graphical representation of the distribution of T‐DNA insertions on tomato chromosomes. Figure S3 Complementation test of 1381ETMM and lyrate mutations. Figure S4 Phenotypic characterization of RNA interference (RNAi) lines for the Solyc11g011960, which was the gene tagged by the T‐DNA insertion in the 2477ETMM line. Table S1 Transformation efficiency in two tomato cultivars. Table S2 Summary of reporter GUS expression. Table S3 Primer sequences used for anchor PCR, genotyping and qRT‐PCR analyses. [file PBI-15-1439-s001.pdf]

A collection of enhancer trap insertional mutants for functional genomics in tomato

Fernando Pérez-Martín[1,+], Fernando J. Yuste-Lisbona[1,+], Benito Pineda[2], María Pilar Angarita-Díaz[2], Begoña García-Sogo[2], Teresa Antón[2], Sibilla Sánchez[2], Estela Giménez[1], Alejandro Atarés[2], Antonia Fernández-Lozano[1], Ana Ortíz-Atienza[1], Manuel García-Alcázar[1], Laura Castañeda[1], Rocío Fonseca[1], Carmen Capel[1], Geraldine Goergen[2], Jorge Sánchez[2], Jorge L. Quispe[1], Juan Capel[1], Trinidad Angosto[1], Vicente Moreno[2] and Rafael Lozano[1,*]

[1] Centro de Investigación en Biotecnología Agroalimentaria (BITAL). Universidad de Almería, 04120 Almería, Spain.

[2] Instituto de Biología Molecular y Celular de Plantas (UPV-CSIC), Universidad Politécnica de Valencia. 46022 Valencia, Spain.

[+] These authors contributed equally to this work.

[*] Corresponding author: rlozano@ual.es

Prof. Rafael Lozano

Dept. Biología y Geología

Universidad de Almería

04120 Almería, Spain

Tel. +34 950 01 5111

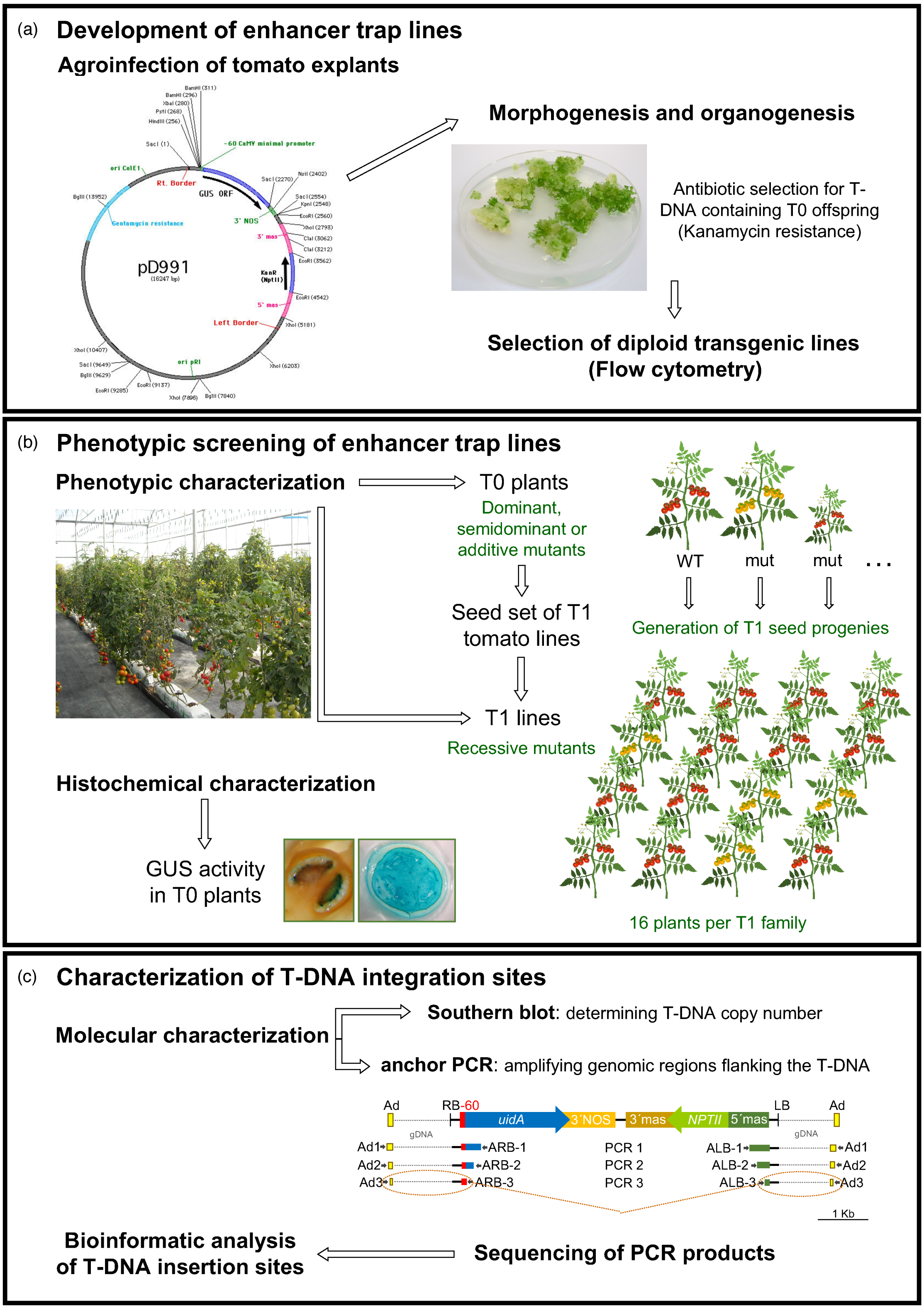

Figure S1. Schematic representation of the T-DNA insertional mutagenesis programme described in this work. (a) Development of the enhancer trap collection using *Agrobacterium*-mediated transformation protocol with the binary vector pD991. (b) Phenotypic and GUS histochemical characterization of enhancer trap lines. (c) Molecular characterization of T-DNA integration sites.

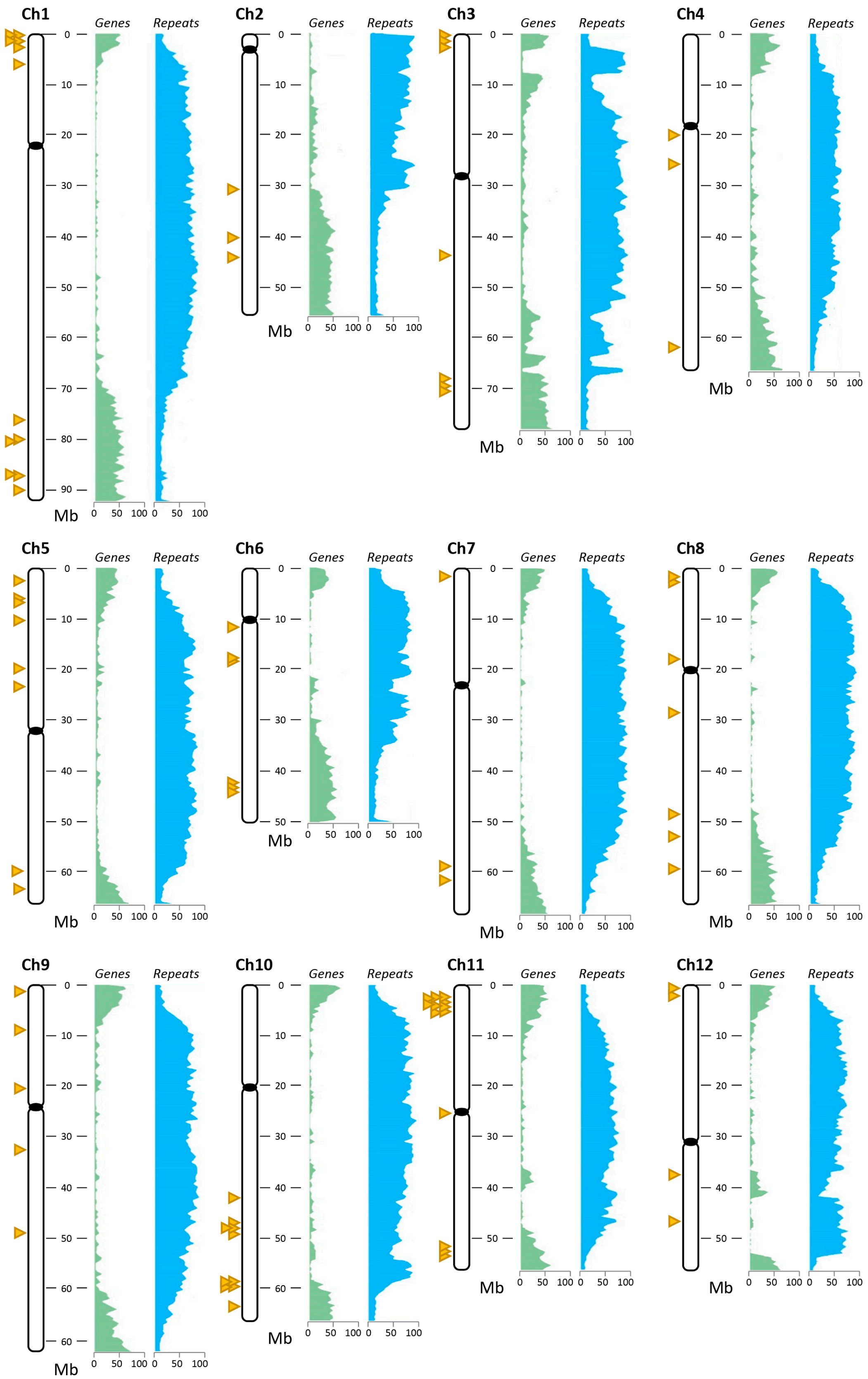

Figure S2. Graphical representation of the distribution of T-DNA insertions (orange arrows) on tomato chromosomes (Ch). Black ovals on the chromosomes indicate the centromere and horizontal lines represent the size in megabases (Mb). Green plots represent the percentage of heterochromatin (% nucleotides per 500kb) and blue plots display the percentage of euchromatin (% nucleotides per 500kb).

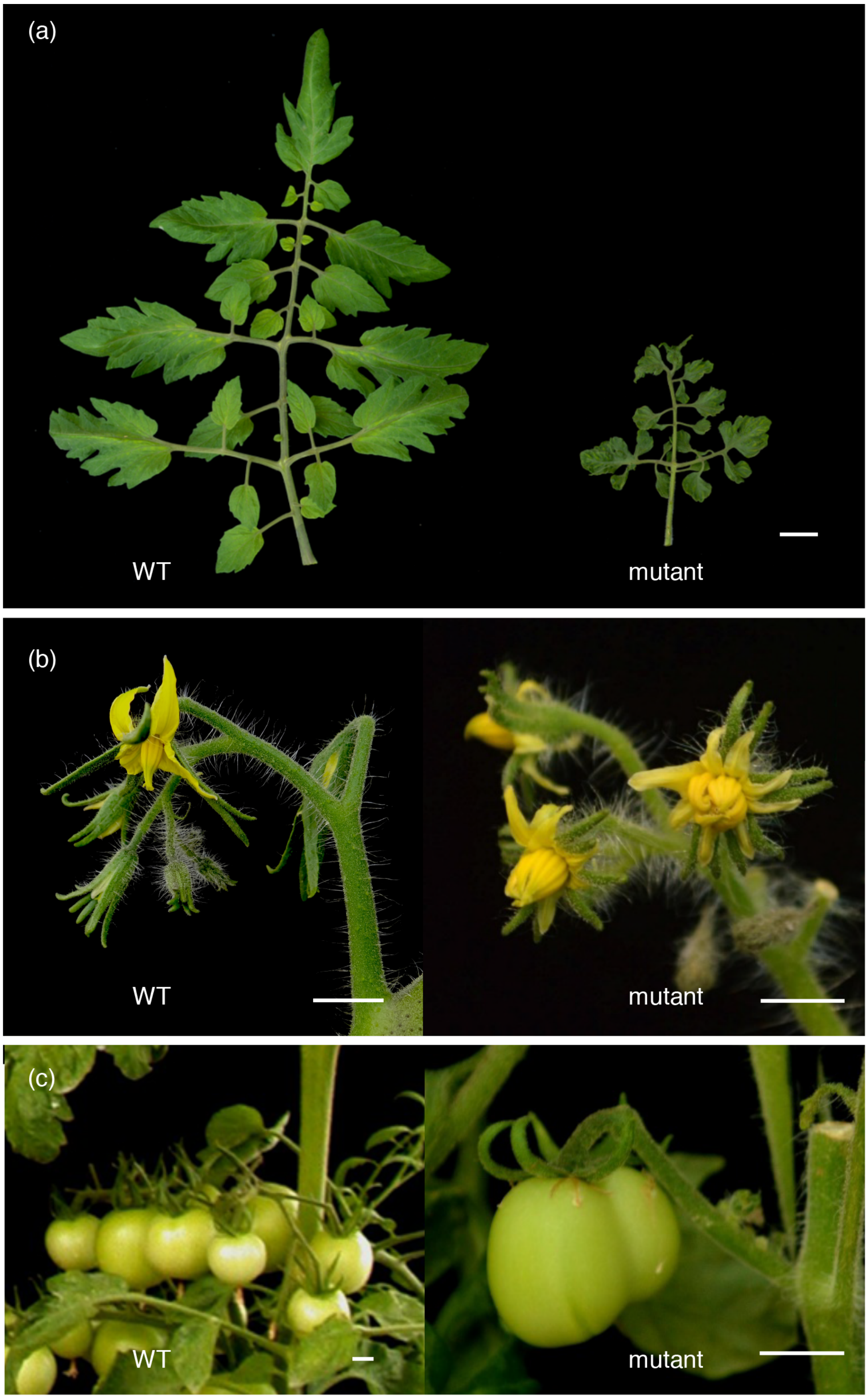

Figure S3. Complementation test of 1381ETMM and *lyrate* mutations. F1 progeny obtained from a cross between wild-type heterozygous plants, one bearing the 1381ETMM mutation (female parent) and the other carrying the *lyrate* mutation (*lyr2*, accession number LA2923, male parent) showed the expected 3:1 segregation (18 WT : 8 mut; $\chi2$ = 0.50, P = 0.46) of wild-type (WT) and mutant phenotypes. Mutant F1 plants were affected in the development of leaves (a), flowers (b) and fruits (c). Scale bar = 5 cm in (a); and 1 cm in (b) and (c).

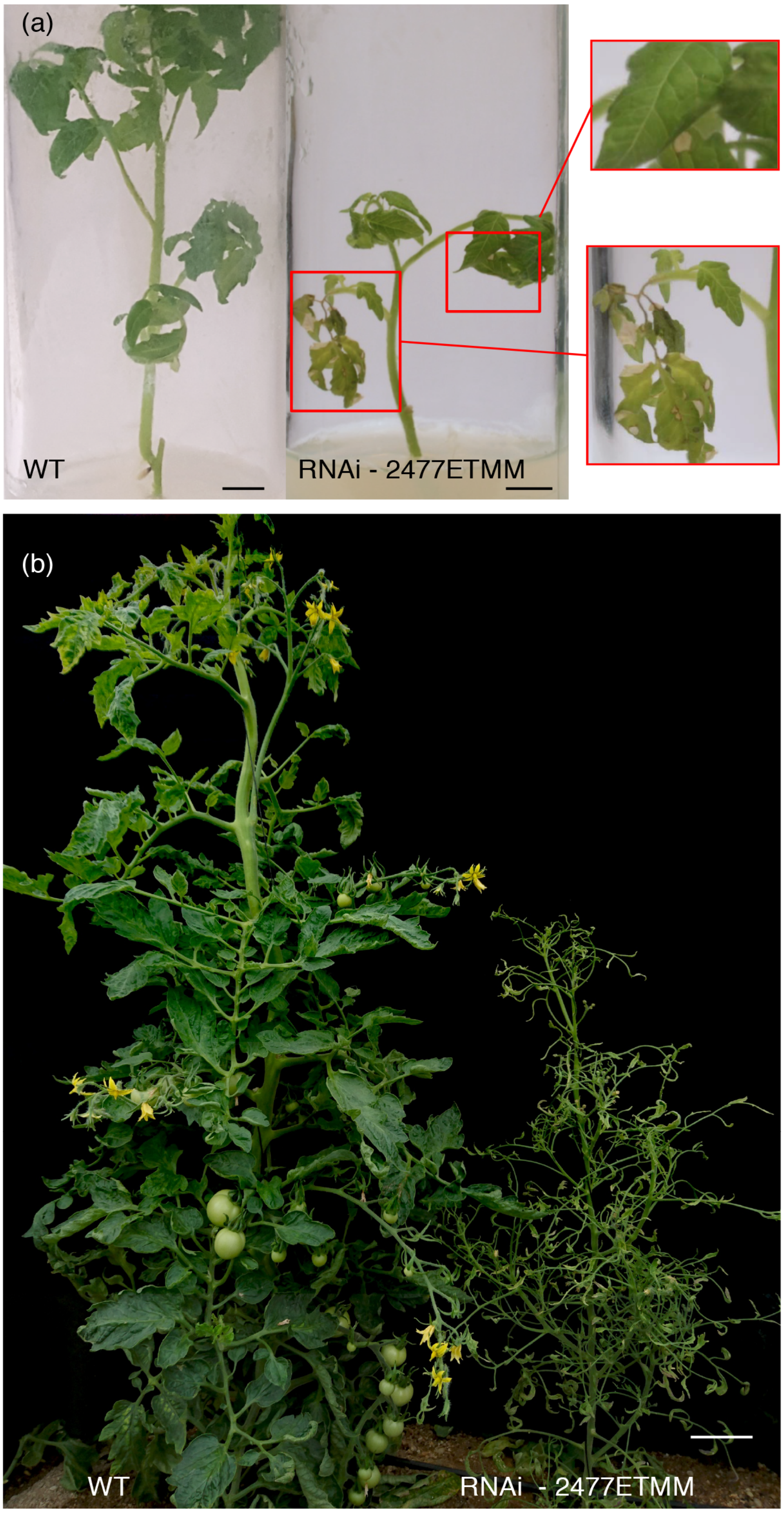

Figure S4. Phenotypic characterization of RNA interference (RNAi) lines for the *Solyc11g011960*, which was the gene tagged by the T-DNA insertion in the 2477ETMM line. Leaves of the T1 RNAi - 2477ETMM plants displayed evident necrosis symptoms and a reduction of plant growth either under *in vitro* (a) or greenhouse (b) conditions, similar to those showed by the 2477ETMM insertional mutant. Scale bar = 1 cm in (a); and 10 cm in (b).

Table S1. Transformation efficiency in two tomato cultivars.

Cultivar	Inoculated explants	Transgenic plants (2*n* + 4*n*)	Transgenic plants (2*n*)	Ratio 2N : 4N	Transf. frequency[a]	% transgenic plants (2*n*)
P73	4200	1816	1021	1 : 0.78	43.2%	56.2%
Moneymaker	18500	6026	4539	1 : 0.33	32.6%	75.3%
Total	22700	7842	5560	1 : 0.41	34.6%	70.9%

[a]Transformation frequency was estimated as the number of independent transgenic events divided by the total number of inoculated leaf explants, then multiplied by 100.

Table S2. Summary of reporter GUS expression.

	Sample	Number of lines showing GUS expression in[a]	Number of lines showing GUS expression restricted to[b]
Vegetative structures	Root	16	4
	Stem	141	23
	Rachis	117	4
	Petiole	151	4
	Leaflet	164	14
Flowers	Sepal	56	1
	Petal	48	3
	Stamen	359	219
	Pistil	158	24
	Stigma	38	4
	Style	43	9
	Ovary	56	7
	Ovule	25	2
Immature fruits	Pericarp	227	78
	Placenta	103	13
	Mucilage	49	6
	Embryo	199	92

[a]Number of lines showing GUS expression in the evaluated tissue.
[b]Number of lines displaying GUS staining restricted to the evaluated tissue.

Table S3. Primer sequences used for anchor PCR, genotyping and qRT-PCR analyses.

A. Primers used for anchor PCR analysis

Primer name	Primer sequence (5'-3')
Ad1	CTAATACGACTCACTATAGGC
Ad2	CTATAGGGCTCGAGCGGC
Ad3	AGCGGCGGGGAGGT
ARB-1	ACAGTTTTCGCGATCCAGAC
ARB-2	GGTCTTGCGAAGGATAGTGG
ARB-3	CTGGCGTAATAGCGAAGAGG
ALB-1	TTGGCGTGTCAGCGTATCTA
ALB-2	ATCGGTCTCAATGCAAAAGG
ALB-3	ATAATAACGCTGCGGACATCTAC

B. Primers used for genotyping analysis

Primer name	Primer sequence (5'-3')
Gt5-F	AAGGAAGCTAGGAATCAACAAGA
Gt5-R	ATTTCTCGGTGAAGGGGTTC
Gt6-F	TGCTCAATGAAGTGGTCGAA
Gt6-R	TTGCAATATTGGTCCCTGAA
Gt11-F	GAAGTGGGGCAAAGTCTTCA
Gt11-R	GAGGCCGGGATCTATCTTTC

C. Primers used for qRT-PCR assays

Primer name	Primer sequence (5'-3')
1381_Fz	CATCCCCAATGCTCATTCTT
1381_Rz	ATGCAGTGAAACCCTCCATC
2477_Fz	ATCCCGCGGAAACTAAGAAG
2477_Rz	GTGCATCCCATTGTTGTTCC
Ubiquitine3_Fz	CACACTTCACTTGGTCTTGCGT
Ubiquitine3_Rz	TAGTCTTTCCGGTGAGAGTCTTCA
